# Microwave-Assisted Dehydrogenative Cross Coupling Reactions in γ-valerolactone with a Reusable Pd/β-cyclodextrin Crosslinked Catalyst

**DOI:** 10.3390/molecules24020288

**Published:** 2019-01-14

**Authors:** Silvia Tabasso, Emanuela Calcio Gaudino, Elisa Acciardo, Maela Manzoli, Agnese Giacomino, Giancarlo Cravotto

**Affiliations:** 1 Department of Chemistry, University of Turin, Via P. Giuria 7, 10125 Turin, Italy; silvia.tabasso@unito.it; 2 Department of Drug Science and Technology and NIS - Centre for Nanostructured Interfaces and Surfaces, University of Turin, Via P. Giuria 9, 10125 Turin, Italy; emanuela.calcio@unito.it (E.C.G.); elisa.acciardo@gmail.com (E.A.); maela.manzoli@unito.it (M.M.); agnese.giacomino@unito.it (A.G.)

**Keywords:** microwaves, C–H activation, heterogeneous catalyst, dehydrogenative cross coupling, GVL

## Abstract

Transition-metal mediated C–H bond activation and functionalization is one of the most straightforward and powerful tools in modern organic synthetic chemistry. Oxidative C–H/C–H coupling reactions between two (hetero)arenes under heterogeneous catalysis may be a valuable means for the production of a plethora of bi(hetero)aryls, and one that adheres to the increasing demand for atom-economic and sustainable chemistry. We have therefore developed a reusable heterogeneous catalytic system, which is based on Pd cross-linked β-cyclodextrin, to perform an efficient microwave-assisted oxidative C–H/C–H cross coupling process between benzothiazoles and methyl thiophene in the presence of green solvents.

## 1. Introduction

The development of environmentally benign and efficient synthetic protocols is still a central goal for research in chemistry [1,2], and transition-metal mediated C–H bond activation is an on-going challenge for organic chemistry. In this context, oxidative C–H coupling between two (hetero)arenes raises considerable issues in terms of both chemo- and regioselectivity in bi(hetero)aryls synthesis as it entails the activation of two different C–H bonds [3,4]. Bi(hetero)aryls are the favored π-conjugated scaffolds for many synthetic intermediates and biologically active molecules also have a wide application in pharmaceutical and material sciences [5,6,7]. The standard approach to bi(hetero)aryls involves palladium-catalyzed cross coupling between two activated substrates, a heteroaryl halide and an organometallic species, as occurs in the Suzuki, Stille, Hiyama, and Negishi coupling reactions (C– X/C–M type) [8,9,10,11]. Since DeBoef [12], and Fagnou [13], first documented Pd(OAc)_2_-catalyzed intermolecular oxidative heteroaryl–aryl cross coupling, a range of unsymmetrical biheteroaryls have been prepared using oxidative cross-coupling reactions [14]. Moreover, its atom economy makes this transformation an active strategy in organometallic catalysis [15]. Although the cross coupling of two simple heteroarenes via double C–H activation is the most straightforward and effective method for the syntheses of these compounds (only a “H_2_” is released), harsh conditions are often required as the energy requirement for aromatic C–H bond dissociation is higher than that for C–X and C–M bond breakage [16]. However, reactivity, regioselectivity, and heteroarene decomposition issues mean that intermolecular heteroarylations still have limited scope. Hu and You [17], Zhang and Li [18], Yamaguchi and Itami [19], and Ofial [20], have recently pushed this approach forward by designing the selective palladium-catalyzed cross dehydrogenative couplings (CDC) of two heteroarenes, thus avoiding the pre-activation step [21]. An efficient method for the synthesis of novel imidazopyridine fused indoles, based on a one-pot sequential Knoevenagel condensation of readily accessible active methylene azoles with *N*-substituted-1H-indole-3-carboxaldehydes, was developed and was followed by palladium/copper-catalyzed intramolecular CDC reactions [22]. More recently, the same catalytic system has been used for the first time for an oxidative C–H/C–H cross coupling between two heteroarenes, with oxygen as the terminal oxidant, in an investigation performed by You et al. [23]. Molecular oxygen (O_2_) may well be the ideal oxidant as it has a low cost and lacks toxic by-products, making it a highly appealing reagent with which to address key “green chemistry” priorities in industry. The benzothiazole moiety is frequently found in compounds of pharmaceutical interest and in materials with optical properties, therefore making the synthesis of biheteroarenes that contain the benzothiazole motif of strategic importance [24,25,26].

The CDC of benzothiazole with thiophenes and thiazoles has been explored by Yang et al. who used dimethylsulfoxide (DMSO) as the solvent and Pd(OAc)_2_ as the catalyst [27]. This protocol followed the paper by You et al. on the coupling of benzimidazoles/benzoxazoles using thiophenes. [17]. A more sustainable dehydrogenative C–C cross-coupling protocol can be efficiently designed using a multi-faceted strategy that combines eco-compatible reaction media, recyclable heterogeneous catalysts, and suitable enabling techniques. This approach involves heterogeneous multiphase reactions in green solvents and in a closed microwave (MW) reactor, which act as key alternatives to conventional protocols. MW dielectric heating can help to develop new, safe, and energy efficient cross-dehydrogenative coupling reactions that may help to satisfy the relentless demand for more highly sustainable chemistry. The benefits of MWs have been well documented in the literature (remarkable reaction time reduction, improved yields, and cleaner reactions than those performed under conventional thermal conditions) [28,29]. Furthermore, since Varma introduced solid catalysts to MW-assisted organic synthesis [30], a broad array of new heterogeneous catalytic applications have been reported [31,32]. Aiming to further increase the sustainability of the process, the search for safer reaction media has recently led to special attention being drawn towards bio-derived solvents [33], which fulfil the green chemistry principles of replacing hazardous solvents with environmentally benign ones. Intensive research activity into biomass conversion [34] has led to the development of several new greener solvents [35,36], including γ-valerolactone (GVL), ethyl lactate, ethyl levulinate, and 2-methyltetrahydrofuran, which may well replace currently used organic solvents. Since it was first suggested by Horváth [37], GVL has been considered a renewable and safe alternative to classical dipolar aprotic solvents for organic synthesis [38,39] and has been used in sustainable C–H functionalization reactions [40,41]. It has recently been reported that GVL provided more benefits than traditional solvents, such as limited metal leaching, when combined with heterogeneous palladium catalysts [42,43].

Our group have recently synthesized a new class of heterogeneous Pd catalysts that are supported on a β-cyclodextrin (β-CD) crosslinked network (the Pd/CβCAT system) [44,45], via the sonochemical reticulation of β-cyclodextrin with hexamethylene diisocyanate (HDI) in the presence of a Pd(II) salt solution [46]. The cheaper and available in bulk β-CD, was preferred to α- and γ-CD because of the comparable catalytic effect already documented in cross-linked CD catalysts. Intermolecular cavities and cross-linking degree strongly affect the catalytic process and metal leaching, minimizing the role of cyclodextrins’ inner cavities size. This Pd/CβCAT catalyst has proven to be particularly efficient in the absorption of MW irradiation, making it an ideal candidate for the synergic combination of the non-conventional enabling technology and heterogeneous catalysis [45,47].

We herein report the first MW-assisted sustainable dehydrogenative C–C cross-coupling procedure for the direct C-2 heteroarylation of benzo[d]thiazole with 2-methylthiophene. The heterogeneous Pd/CβCAT catalytic system gave corresponding products in good yields in the presence of green solvents. Furthermore, the catalyst was recycled and reused for five runs.

## 2. Results and Discussion

### 2.1. Catalyst Preparation and Characterization

**Scheme 1:**
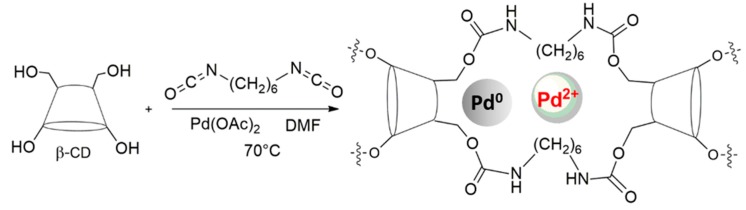
Preparation of poly-βCD/Pd nanoparticles (Pd/CβCAT).

Heterogeneous catalysis is a valid alternative for metal catalyzed reactions and thus furthers the search for sustainable processes. Indeed, the ability to incapsulate a metal within a solid framework dramatically reduces metal leaching into solution, making the catalyst reusable and recyclable. A poly β-CD/Pd catalyst that is crosslinked with hexamethylenediisocyanate (HDI) was prepared according to a previously described procedure (Scheme 1) [46], with this goal in mind. The amount of HDI was optimized in order to obtain a reticulated compact gel.

**Figure 1:**
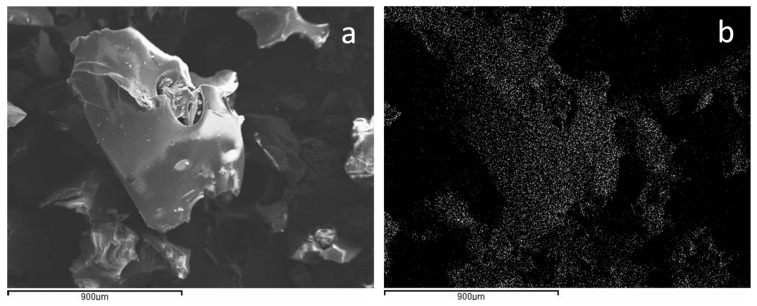
Scanning electron microscope (SEM) image of the Pd/CβCAT catalyst (section **a**) and corresponding EDS Pd mapping (section **b**).

The product was then analyzed by Inductively Coupled Plasma-Optical Emission Spectrometry (ICP–OES), revealing an average metal content of 27.97 ± 1.20 g/kg. Palladium was widely and uniformly dispersed within the CD matrix, as demonstrated by the metal mapping performed using the Energy Dispersive (EDS) probe, as reported in section b of Figure 1, together with the corresponding Scanning Electron Microscopy (SEM image (section **a**)).

**Figure 2:**
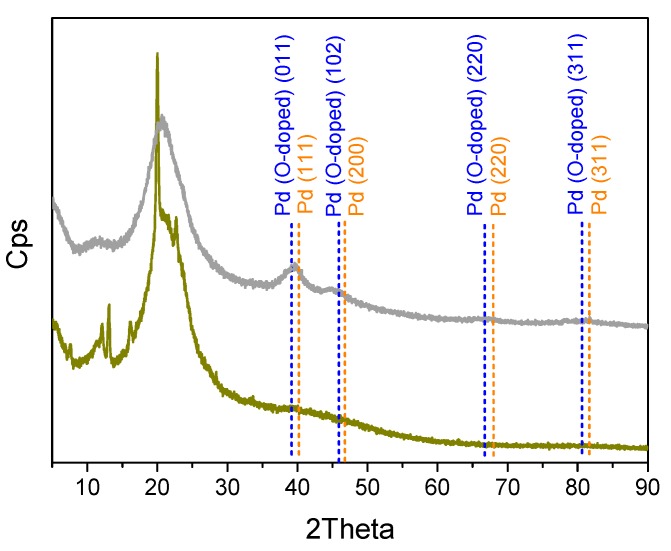
Powder X-ray diffraction (XRD) patterns of poly-βCD (ochre curve), and of the fresh (grey curve) Pd/CβCAT catalyst.

The structure of the obtained materials was investigated using X-ray diffraction (XRD) and the results are shown in Figure 2. The narrow peaks in the XRD pattern of β-CD (ochre curve) that are due to crystalline β-CD [48,49], overlap with a broad peak that is related to the presence of an amorphous phase, which is possibly caused by the presence of crosslinked hexamethylene diisocyanate moieties. These peaks were not present in the pattern collected from Pd/CβCAT (grey curve), where the broad amorphous-phase peak was observed along with two less intense peaks in the 39 to 45 2Theta range. These features highlight the re-organization of the crosslinked β-CD polymer crystalline phase into a less ordered material upon the addition of Pd, along with the presence of a small amount of highly dispersed metal nanoparticles, as revealed by the broad peaks at 2Theta 39.7° and 45°. Interestingly, the position of these peaks was intermediate with respect to that of Pd in the cubic phase (the main peak positions are signaled by dashed orange lines, JCPDS file number 00-005-0681), and that related to *O*-doped palladium in the hexagonal phase (the main peak positions are signaled by dashed blue lines, JCPDS file number 01-072-0710). For the sake of comparison, the standard JCPDS stick patterns of both Pd phases have been reported in the Supporting Information. The above features could be an indication that the Pd nanoparticles, due to their small size, are oxidized at the surface due to air exposition. Indeed, a rough estimation of the particle dimension can be done by applying the Scherrer’s equation to the peak at 39.7°:

(1) $$\tau =\frac{k}{\beta cos\theta }=2.97\;\mathrm{nm}$$

where *τ* represents the mean size of the crystalline domains, *K* is the dimensionless shape factor, with a value of 0.9, *λ* is the X-ray wavelength (for Cu, λ = 0.541 Å), *β* is the line broadening at half the maximum intensity (FWHM) after subtracting the instrumental line broadening, and *θ* is the Bragg angle. The obtained value (2.97 nm) is in agreement with the previous hypothesis.

**Figure 3:**
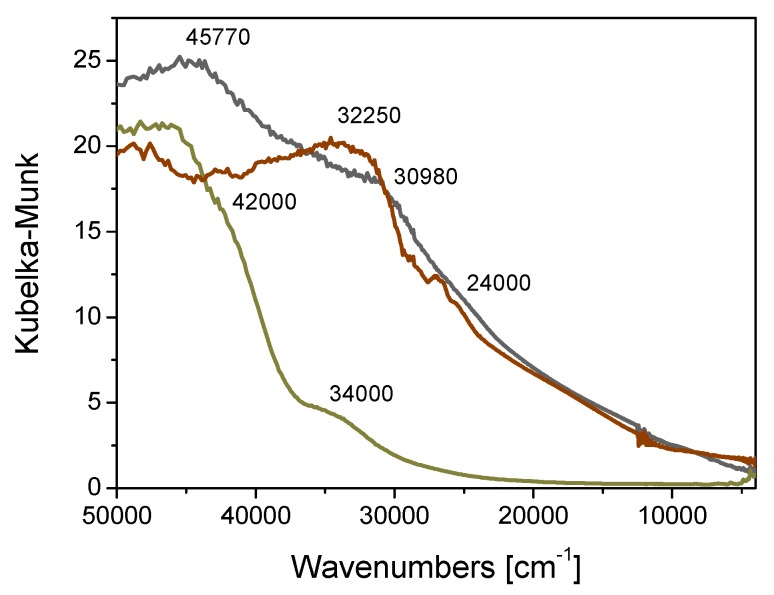
DR UV–Vis spectra of crosslinked βCD (ochre curve), fresh (grey curve) and used (after the 6th run) Pd/CβCAT catalyst (brown curve).

The Pd oxidation state was evaluated using diffuse reflectance UV–Vis spectroscopy and the results are reported in Figure 3. In particular, the shoulder at 24,000 cm^−1^, which is present in the catalyst spectrum (grey curve) and not in that of crosslinked β-CD alone (ochre curve), can be ascribed to the d–d transition of Pd^2+^ species [50].

**Figure 4:**
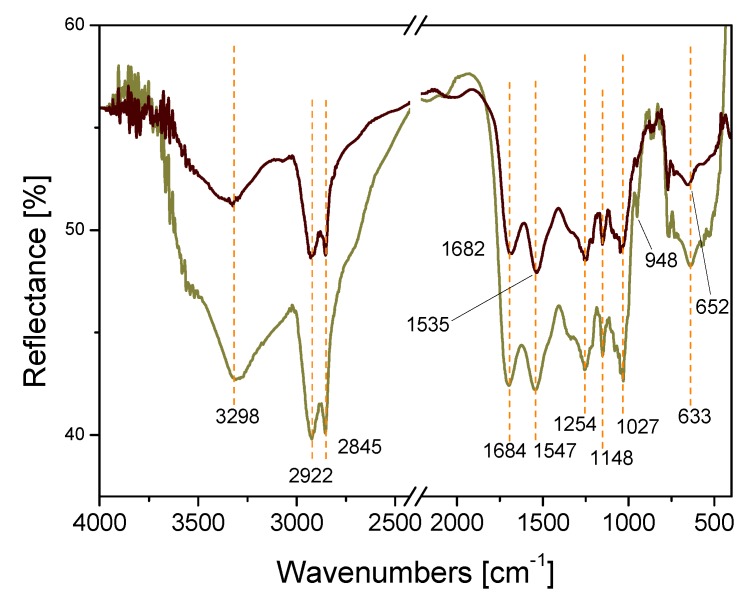
Diffuse reflectance Fourier transform infrared spectroscopy (DRIFT) spectra of crosslinked βCD (ochre curve), and the Pd/CβCAT catalyst (grey curve). The dashed orange lines serve as eyeguides.

A comparison of the diffuse reflectance Fourier transform infrared spectroscopy (DRIFT) spectra of crosslinked β-CD (ochre curve), and the Pd/CβCAT catalyst (grey curve) is shown in Figure 4. DRIFT spectroscopy was used to identify specific functional groups and their modification upon Pd^2+^ insertion. In the β-CD DRIFT spectrum, the bands at 3298, 1684, 1547, and 1148 cm^−1^ correspond to the O–H stretching vibration, the C=O stretching of the carboxyl groups, the C–O stretching vibration, and the N–H bending vibration, respectively [49,51,52]. The bands observed at 1254 and 1027 cm^−1^ can be ascribed to the symmetric and antisymmetric glycosidic C–O–C vibrations in the ether groups [49,51,52]. The peaks at 2923 and 2853 cm^−1^ represent the -CH_2_ symmetric and asymmetric stretching vibrations, respectively. The absorption band at 948 cm^−1^ can be assigned to the R-1,4-bond skeleton vibration of β-cyclodextrin [49]. The peak at 633 cm^-1^ is related to O–H bending (out-of-plane).

A qualitative comparison between the DRIFT spectra of crosslinked βCD (ochre curve) and the Pd/CβCAT catalyst (grey curve) was performed. The following differences were observed between the spectrum of Pd/CβCAT and that of crosslinked βCD: (i) The broad band at 3298 cm^−1^ show reduced relative intensity and a change in shape, (ii) the peaks at 1684 and 1547 cm^−1^ are red shifted in position (to 1682 and 1535 cm^−1^, respectively), (iii) the peak at 1027 cm^−1^ has lower relative intensity, and (iv) the band at 633 cm^−1^ is noticeably blue shifted to 652 cm^−1^.

These spectroscopic features may indicate that fewer OH group intramolecular interactions occur (band at 3298 cm^−1^) and that C–O–C ether groups present decreased mobility (peak at 1027 cm^−1^) upon Pd^2+^ insertion. Furthermore, the blue shift of the band that is assigned to the out-of-plane bending of OH groups (at 633 cm^−1^) possibly indicates that the tangles formed by the polymeric chains after metal addition are larger. Moreover, the red shift of the peaks at 1684 and 1547 cm^−1^ indicate that these groups are perturbed and therefore possibly involved in the interaction with the metal cation, whereas the N–H groups do not seem to be perturbed by the embedding of Pd^2+^.

### 2.2. Microwave-Assisted Dehydrogenative Cross Coupling Reactions

The polar nature of the cyclodextrin network makes Pd/CβCAT very sensitive to dielectric heating, and therefore particularly suitable for MW-assisted reactions. This feature is enhanced by the presence of the embedded cation. The synergistic combination of MW and this heterogeneous catalyst has already been proven to be successful in Heck and Suzuki couplings [44]. This reason and the desire to find more sustainable processes for the synthesis of heterocycles drove us to further investigate this approach and apply it to the oxidative cross-coupling reaction between benzo[d]thiazole and 2-methythiophene. This reaction has already been described as occurring under conventional heating and homogeneous catalysis in *N*,*N* dimethylacetamide (DMA) and DMSO [27], using AgNO_3_ as the oxidant in the presence of a number of ligands. Under these conditions, the highest product yield (69%) was described after 10 h at 110 °C. This protocol lacks sustainability for many reasons, and thus we have proposed an alternative and more eco-friendly process that is based on: (i) The use of heterogeneous, instead of homogeneous, catalysis; (ii) the replacement of DMSO with green solvents; (iii) the use of non-conventional dielectric heating, with the aim of reducing reaction time and the formation of by-products.

**Scheme 2:**
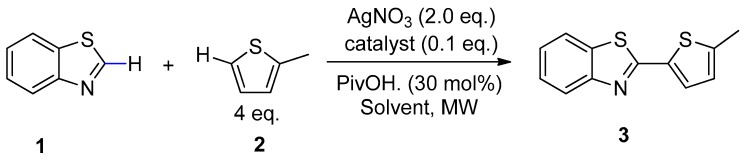
Microwave (MW)-assisted oxidative cross-coupling reaction between benzo[d]thiazole and 2-methythiophene.

The peculiar MW reactor used in this work is equipped with a pressure-control system and a multiple-position rack and can screen the reaction conditions of parallel reactions runs, while also further reducing the overall optimization process time. Our group have recently investigated the MW-assisted C–H arylation of thiophenes in GVL [39], which was used as an alternative to DMA. In that work, pivalic acid (PivOH) was used as the ligand as it can promote the concerted metalation/deprotonation process (CMD), and we therefore decided to use it in the CDC reaction (Scheme 2) with a variety of biomass-derived solvents. Ethyl lactate, ethyl levulinate and GVL, which have recently been used as alternative green reaction mediums for C–H functionalization processes [53], were compared to DMA.

As the main scope of these preliminary studies was to choose the most suitable solvent for the catalytic system, AgNO_3_ was used as the oxidant and the results were compared with those obtained under homogeneous catalysis (with Pd(OAc)_2_ as the catalyst). It is worth nothing that MW significantly promoted the reaction. In fact, yields are much higher (100% in this work vs 36% according to Yang et al. [27], Table 1, entry 1) under the same conditions as those indicated in the literature ((Pd(OAc)_2_ in the presence of DMA and PivOH).

In regard to the green solvents, ethyl lactate and ethyl levulinate favored the formation of the homocoupling product, both under homogeneous and heterogeneous catalysis (Table 1, entries 3 and 4). The best results were achieved using GVL. This is not surprising as GVL can be considered a valid alternative to common aprotic solvents thanks to its similar polarity. Furthermore, its high dielectric constant (36.47) makes it particularly prone to interaction with MW. It is worth noting that GVL gave better results when used with Pd/CβCAT than with Pd(OAc)_2._ (Table 1, entry 2). It is well known that Pd(OAc)_2_ gives rise to insoluble (Pd black) nanoparticles in the absence of ancillary ligands at temperatures above 120 °C [39]. DMA is more efficient than GVL in coordinating the palladium cations, thus keeping them in the homogeneous phase and giving higher product yields. However, the differences between DMA and GVL become insignificant when Pd/CβCAT is used, as the stability of the active palladium species can be ascribed to the catalyst network, under heterogeneous conditions. This may explain why the same yield is obtained when using the two solvents together with Pd/CβCAT. The same reaction in the presence of Pd-free β-cyclodextrin network gave no reagents conversion (data not reported).

Carboxylic acids, such as PivOH, are usually added to suppress undesired homocoupling reactions and favor the deprotonation step [14]. However, stronger acids, such as trifluoroacetic acid (TFA), gave lower yields as well as considerable amounts of the homocoupling product. This is the main product (83%, Table 2, entry 1) of the heterogeneous catalysis in DMA, while there is no difference in terms of yields between the two catalysts in GVL (Table 2, entry 2). Even lutidine, which can be used in these reactions, mainly afforded the homocoupling product in the presence of Pd/CβCAT (Table 2, entry 3), and its use in GVL was not therefore investigated any further.

In order to increase the sustainability of the process, molecular oxygen was investigated for use as the oxidant, as water is the only stoichiometric by-product formed. C–H alkenylation in GVL using oxygen as the sole oxidant and [RuCl_2_(p-cymene)]_2_ with potassium acetate (KOAc) has recently been described [54]. However, in the CDC of benzothiazole and 2-methylthiophene, product yields were considerably lower than when AgNO_3_ was used (Table 3, entries 1 and 2). This result suggests that this reaction may proceed via reductive elimination, rather than β-hydride elimination, as the latter is usually compatible with O_2_ as the oxidant [55]. As Ag(I) can promote C–C reductive elimination from Pd, [56,57] its replacement with molecular oxygen probably requires ancillary ligands that can play the same role in that step and promote aerobic catalytic turnover [56]. No significant improvement in results was achieved in DMA even when other oxidants, such as K_2_S_2_O_8_ (Table 3 entry 3) and Ag_2_O (Table 3 entry 4) were used in combination with molecular oxygen. For this reason, we decided not to use these oxidant combinations in GVL in any further experiments. Only the addition of AgNO_3_ to O_2_ led to good coupling product yields in DMA (Table 3, entry 5), both with Pd(OAc)_2_ and Pd/CβCAT. However, the results were worse in GVL (Table 3, entry 6), meaning that the use of AgNO_3_ alone was therefore confirmed as the best oxidant option (Table 3, entries 9 and 10).

In order to further investigate the mechanism of this CDC reaction, 2,2,6,6-tetramethylpiperidinyl-1-oxy (TEMPO, 30% mol) was added as a radical scavenger to the reaction system under the optimized conditions with Pd/CβCAT as the catalyst. The formation of the product (**3**) was strongly inhibited as only the homocoupling product was observed (Table 3, entry 11). It therefore appears that a radical pathway may be involved in the process. This hypothesis is corroborated by the ability of silver (I) salts to promote the one-electron oxidative cleavage of the Pd–C bond to give a heteroaryl radical [58]. These results, coupled with the significant influence of pivalic acid on the reaction yields, supports the mechanism, a CMD pathway followed by a radical transfer, that was proposed by Yang et al. [27] However, the elucidation of the entire mechanism remains a challenge.

### 2.3. Catalyst Reusability

**Figure 5:**
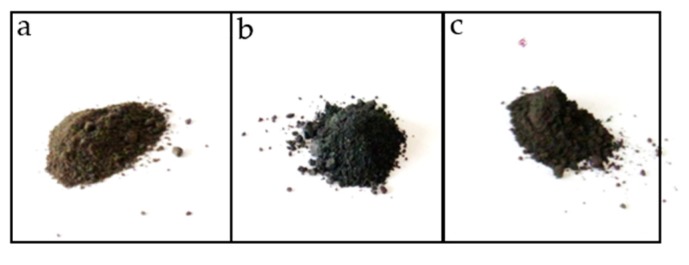
Comparison of the digital images taken of fresh Pd/CβCAT (section **a**), and that recovered after the second (section **b**) and after sixth runs (section **c**).

**Scheme 3:**
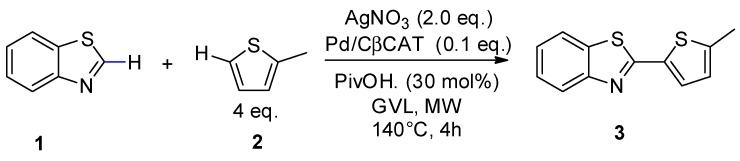
MW-assisted dehydrogenative coupling conditions for recycling tests.

The advantages of a heterogeneous catalyst lie in its recovery and reusability, which can either facilitate a reduction in separation steps, or the abatement of waste, and therefore favor process sustainability. The solid Pd/CβCAT was recovered from the crude reaction via simple filtration under vacuum, and washed with water, methanol, and acetone. The catalyst was then reused in subsequent reactions under the optimized conditions (Scheme 3). The recovered solids were of similar appearance (Figure 5).

As shown in Table 4, the Pd/CβCAT catalyst can be used for five consecutive runs of the reaction between **1** and **2** with a limited reduction in product yield (Table 4).

Furthermore, the amount of metal in the recycled catalyst was measured after the second run by ICP–OES analysis, and was found to be the same as in the fresh catalyst.

**Figure 6:**
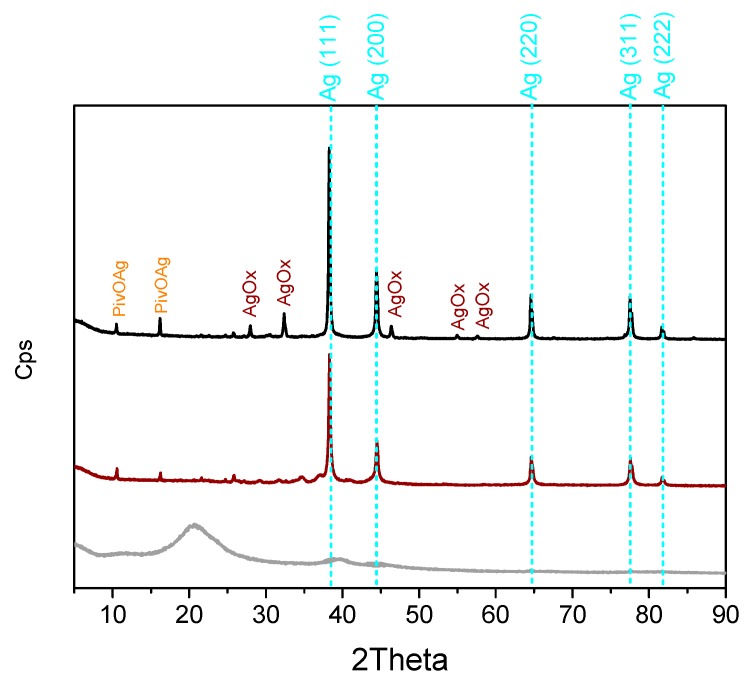
Powder XRD patterns of the fresh (grey curve), recovered after the second run (brown curve) and after the sixth run (black curve) Pd/CβCAT catalyst.

The XRD patterns of the catalyst that was used and recovered after the second (black curve) and the sixth (brown curve) runs are reported in Figure 6. When compared to the pattern of the fresh sample (grey curve, already shown in Figure 2), several new peaks, whose intensity increases with the number of the catalytic cycles, can be seen. Such peaks are related to the presence of a mixture of metallic Ag in the cubic phase (that have been indexed and highlighted by dashed cyan lines, JCPDS file number 00-001-1167), and of silver oxides (Ag_2_O, JCPDS file number 00-003-0796; AgO, JCPDS file number 00-022-0472; and Ag_3_O_4_, JCPDS file number 03-065-9750, peaks denoted with “AgOx”). For the sake of comparison, the standard JCPDS stick pattern of the Ag(0) phase has been reported in the Supporting Information). Furthermore, the peaks at low 2Theta angles can be tentatively assigned to crystalline silver pivalate (denoted as PivAg, JCPDS file number 00-053-1564).

We propose that the deactivation of the Pd/CβCAT catalyst may be due to the presence of silver crystalline nanoparticles that are a result of the agglomeration of metallic silver atoms during the reaction and produced by the reduction of Ag^+^ (added as AgNO_3_) to Ag^0^. These nanoparticles gradually accumulate at the catalyst surface with subsequent catalytic cycles, and possibly block the palladium active sites, resulting in the yield drop observed at the sixth run. This hypothesis is corroborated by the presence of the component at 24,000 cm^−1^ in the spectrum collected on the Pd/CβCAT catalyst after the sixth run (brown curve in Figure 3), which indicates that Pd^2+^ species are still present.

## 3. Materials and Methods

### 3.1. Catalyst Preparation

Pd(OAc)_2_ (200 mg, 0.90 mmol, Sigma Aldrich, Saint Louis, MI, USA) and β-CD (1 g, 0.78 mmol, Wacker Chemie, Munich, Germany) were dissolved in DMF (Sigma Aldrich, Saint Louis, MO, USA) (4 mL). Hexamethylene diisocyanate (HDI) (2.8 mL, 17.4 mmol, Sigma Aldrich, Saint Louis, MO, USA) was then added portion-wise, and the reaction mixture was kept at 70 °C for 30 min. The compact gel was crushed and washed with water (50 mL), methanol (50 mL), and acetone (50 mL). The product was filtered on a sintered glass Buchner funnel and dried overnight under vacuum at 75 °C to produce a brownish powder (with Pd (II). The average metal content in the various crosslinked CD catalysts was analyzed using ICP-OES (Perkin Elmer, Norwalk, CT, USA).

### 3.2. General Procedure for the MW-assisted Dehydrogenative Cross Coupling Reaction

Benzo[d]thiazole (110 μL, 1 mmol) and 2-methythiophene (387 μL, 4 mmol) were dissolved in 3 mL of solvent in a flask. After the addition of the catalyst (10 mol% of Pd), the mixture was heated to 140 °C under stirring (450 rpm) in the presence of the oxidant and ligand for 4 h in a multimode MW reactor (SynthWAVE, Milestone Srl, Italy; MLS GmbH, Germany) equipped with a multiple safe gas-loading system, under either a N_2_ or O_2_ atmosphere. After cooling, the crude reaction was filtered on a sintered glass Buchner funnel, 100 µL of the reaction mixture was then diluted with ChCl_3_ (1 mL) and the resulting solution was analyzed using GC-MS (Agilent Technologies, Santa Clara, CA, USA). Water (3 mL) was then added as the antisolvent to the remaining crude reaction and the product was recovered via filtration and washed with water (2 mL), dried under vacuum, and purified using column chromatography (petroleum ether/ethyl acetate = 7/3). The product was further characterized by ^1^H-NMR and ^13^C-NMR (Jeol, Akishima, Tokyo, Japan). Related details and spectra are given in the Supplementary Information File (SI-II).

### 3.3. General Procedure for Catalyst Recycling

The catalyst recovered after the filtration of the crude reaction mixture was washed with water (20 mL), acetone (20 mL), and methanol (20 mL), dried under vacuum at room temperature overnight and room temperature, and reused in the next runs under the same optimized reaction conditions. The conversions obtained after the recycling tests were measured by GC-MS. Catalyst structure and Pd loading were also analyzed after recycling.

The total Pd content was determined using ICP–OES with a Perkin Elmer Optima 7000 (Perkin Elmer, Norwalk, CT, USA) spectrometer via standard addition.

## 4. Conclusions

Pd/CβCAT has proven itself to be a valid heterogeneous catalyst for the MW-assisted dehydrogenative cross coupling of benzo[d]thiazole and 2-methylthiophene. The polar nature of its cyclodextrin network means that it is a good MW absorber, and thus provides high yields in reduced reaction times. Furthermore, it was more efficient than Pd(OAc)_2_ when the reaction was performed in the biomass-derived solvent, GVL. Besides the reaction itself, which avoided the synthesis of organometallic intermediates, the sustainability of the process was enhanced by the proven recyclability of the catalyst and the use of a green solvent, while no metal leaching was observed.

## Figures and Tables

**Table 1:** Solvent screening for the dehydrogenative cross-coupling reaction ^a^.

Entry	Solvent	Yield (%) ^b^	
		Pd(OAc)_2_	Pd/CβCAT
1	DMA	100 (89) ^c^	95 (56) ^c^
2	GVL	76 (46) ^c^	95 (43) ^c^
3	Ethyl lactate	60 (40 *homo*)	57 (42 *homo*)
4	Ethyl levulinate	34 (65 *homo*)	15 (58 *homo*)

^a^ Reaction conditions: 140 °C, 4 h. ^b^ Determined by GC-MS (yields are based on area percentage). ^c^ The yields obtained after two hours can be seen in brackets.

**Table 2:** Cross dehydrogenative couplings (CDC) using a variety of ligands ^a^.

Entry	Ligand ^b^	Solvent	Yield (%) ^c^	
			Pd(OAc)_2_	Pd/CβCAT
1	TFA	DMA	46 (53 *homo*)	16 (83 *homo*)
2	TFA	GVL	33 (67 *homo*)	29 (66 *homo*)
3	Lutidine	DMA	67 (32 *homo*)	23 (72 *homo*)

^a^ Reaction conditions: 140 °C, 4 h. ^b^ 30 mol%. ^c^ Determined by GC-MS (yields are based on area percentage).

**Table 3:** Screening of oxidants in the CDC reaction ^a^.

Entry	Oxidant	Solvent	Yield(%) ^b^	
			Pd(OAc)_2_	Pd/CβCAT
1	O_2_ 15 bar	DMA	31	41
2	O_2_ 15 bar	GVL	21	9
3	K_2_S_2_O_8_ + O_2_ 15 bar	DMA	0	0
4	Ag_2_O (1eq.) + O_2_ 15 bar	DMA	0	0
5	AgNO_3_ (1eq.) + O_2_ 15 bar	DMA	89	80
6	AgNO_3_ (1eq.) + O_2_ 15 bar	GVL	53	54
7	Ag_2_O (2eq.)	DMA	18	Traces
8	Ag_2_O (2eq.)	GVL	15	Traces
9	AgNO_3_ (2eq.)	DMA	100	95
10	AgNO_3_ (2eq.)	GVL	76	95
11 ^c^	AgNO_3_ (2eq.)	DMA	n.d.	0 (98 *homo*)

^a^ Reaction conditions: 140 °C, 4 h. ^b^ Determined by GC-MS (yields are based on area percentage). ^c^ TEMPO (30%mol) was added to the reaction mixture.

**Table 4:**
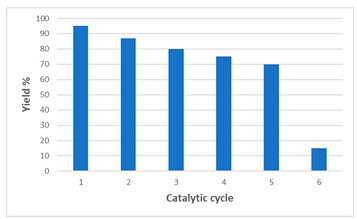
Recycling tests.

Run	Yield ^a^
1	95
2	87
3	80
4	75
5	70
6	15

^a^ Determined by GC-MS (yields are based on area percentage).

## Supplementary Materials

Supplementary Materials are available online. Catalyst instrumentation details, JCPDS stick patterns of Pd(0) and Ag(0), Spectral information, Figure S1: ^1^H-NMR spectrum of 2-(5-methylthiophen-2-yl)benzo[d]thiazole, Figure S2: ^13^C-NMR Spectrum of 2-(5-methylthiophen-2-yl)benzo[d] thiazole.
