# Supplementary material for: Microwave-Assisted Dehydrogenative Cross Coupling Reactions in γ-valerolactone with a Reusable Pd/β-cyclodextrin Crosslinked Catalyst
Source: Molecules. 2019 Jan 14;24(2):288. doi: 10.3390/molecules24020288 (PMC6359118; doi:10.3390/molecules24020288)
Supplement: Supplementary file 1 [file molecules-24-00288-s001.pdf]

Supporting information

Microwave-Assisted Dehydrogenative Cross Coupling Reactions in γ-valerolactone with a reusable Pd/β-cyclodextrin crosslinked catalyst

Silvia Tabasso[1], Emanuela Calcio Gaudino [2], Elisa Acciardo[2], Maela Manzoli[2], Agnese Giacomino[2], and Giancarlo Cravotto[2,*]

[1] Department of Chemistry, University of Turin, Via P. Giuria 7, Turin 10125, Italy
[2] Department of Drug Science and Technology and NIS - Centre for Nanostructured Interfaces and Surfaces, University of Turin, Via P. Giuria 9, Turin 10125, Italy

* Correspondence: giancarlo.cravotto@unito.it; Tel.: +39-011-670-7183

Catalyst Instrumentation Details [Supporting Information (SI–I)]

Scanning Electron Microscopy (SEM) images were acquired on a ZEISS EVO 50 XVP microscope with a LaB_6 source, operating at 10 kV and equipped with detectors for both secondary electron and back scattered electron collection. The samples were sputtered with a gold layer (ca. 10 nm thickness, Bal-tec SCD050 sputter coater) prior to examination. Gold coating thickness had no influence on the observed morphology.
X-ray powder diffraction (XRD) patterns were collected with a PW3050/60 X'Pert PRO MPD diffractometer from PANalytical working in Bragg–Brentano geometry, using as a source the high-powered ceramic tube PW3373/10 LFF with a Cu anode (λ= 0.541 Å) equipped with a Ni filter to attenuate K_β. Scattered photons were collected by a real time multiple strip (RTMS) X'celerator detector. Data were collected in the $5° \leq 2\theta \leq 90°$ angular range, with 0.02° 2θ steps.
Diffuse reflectance UV-Vis-NIR spectra were run on a Varian Cary 5000 spectrophotometer, working in the range of wavenumbers 190-2500 nm. UV-Vis-NIR spectra are reported in the Kubelka-Munk (K-M) function:

$$\mathbf{F(R_\infty) = \frac{(1 - R_\infty)^2}{2R_\infty} = \frac{K}{s}}$$

where R_∞ is the reflectance of an "infinitely thick" layer of the sample, K is the molar absorption coefficient and s is the scattering coefficient. $F(R_\infty)$ is usually termed the remission or K-M function. All spectra were acquired in air and the samples were examined without any preliminary activation.
Diffuse reflectance Fourier transform Infrared spectroscopy (DRIFT) measurements were carried out by using a Spectrum BX FT-IR System (Perkin Elmer) in the range of 4000–400 cm^{-1}.

Experimental Section [Supporting Information (SI–I)]

All chemicals and reagents required for the reaction were purchased from commercial suppliers used without any further purification. β-CD was kindly provided by Wacker Chemie (Munich, Germany).
Product purification was performed by flash chromatography on Silica gel (Merck Kieselgel 60 (0,063-0,200mm)).
NMR Jeol ECZ-R (600 MHz and 150 MHz for ^1H and ^{13}C, respectively) at 25 °C. Chemical shifts (δ) of ^1H NMR and ^{13}C NMR spectra are reported in parts per million (ppm) relative to residual solvent signals ($CHCl_3$ in $CDCl_3$: δ = 7.26 ppm for ^1H and $CDCl_3$: δ = 77.04 ppm for ^{13}C NMR), J values are given in Hz.
The purity of the reaction product was confirmed by TLC using aluminium plates coated with silica gel (Merck Kieselgel 60 F254) which were visualized by UV inspection.

GC-MS analyses were carried out in a gas chromatograph Agilent 6890 (Agilent Technologies, USA), fitted with a mass detector Agilent Network 5973, which used a capillary column that was 30 m long and had an i.d. of 0.25 mm and a film thickness of 0.25 mm. GC conditions were an injection split of 1:20, injector temperature of 250 °C, and detector temperature of 280 °C. The gas carrier was helium (1.2 mL min^{-1}), and the temperature program was from 70 °C (2 min) to 300 °C at 5 °C min^{-1}.
GC-MS analyses were performed in a GC Agilent 6890 (Agilent Technologies - USA) which was fitted with a mass detector Agilent Network 5973, using a HP-5 column (30 m long capillary column, i.d of 0.25 mm and film thickness 0.25 μm.

Spectral Information [Supporting Information SI–II]

2-(5-methylthiophen-2-yl)benzo[d]thiazole (**3**). ^{1}H-NMR ($CDCl_3$, 600 MHz) δ = 7.82 (1H, ddd, J=8.2, J=1.2, J=0.6); 7.63 (1H, ddd, J=8.0, J=1.3, J=0.6); 7.27–7.25 (2H, m); 7.15 (1H, ddd, J=8.0, J=7.3, J=1.2); 6.59 (1H, dq, J=3.4, J=1.1); 2.35 (3H, d, J=1.0).

^{13}C-NMR (150 MHz) δ = 161.73; 153.80; 144.82; 134.94; 134.60; 128.99; 126.58; 126.40; 125.06; 122.83; 121.47; 15.74

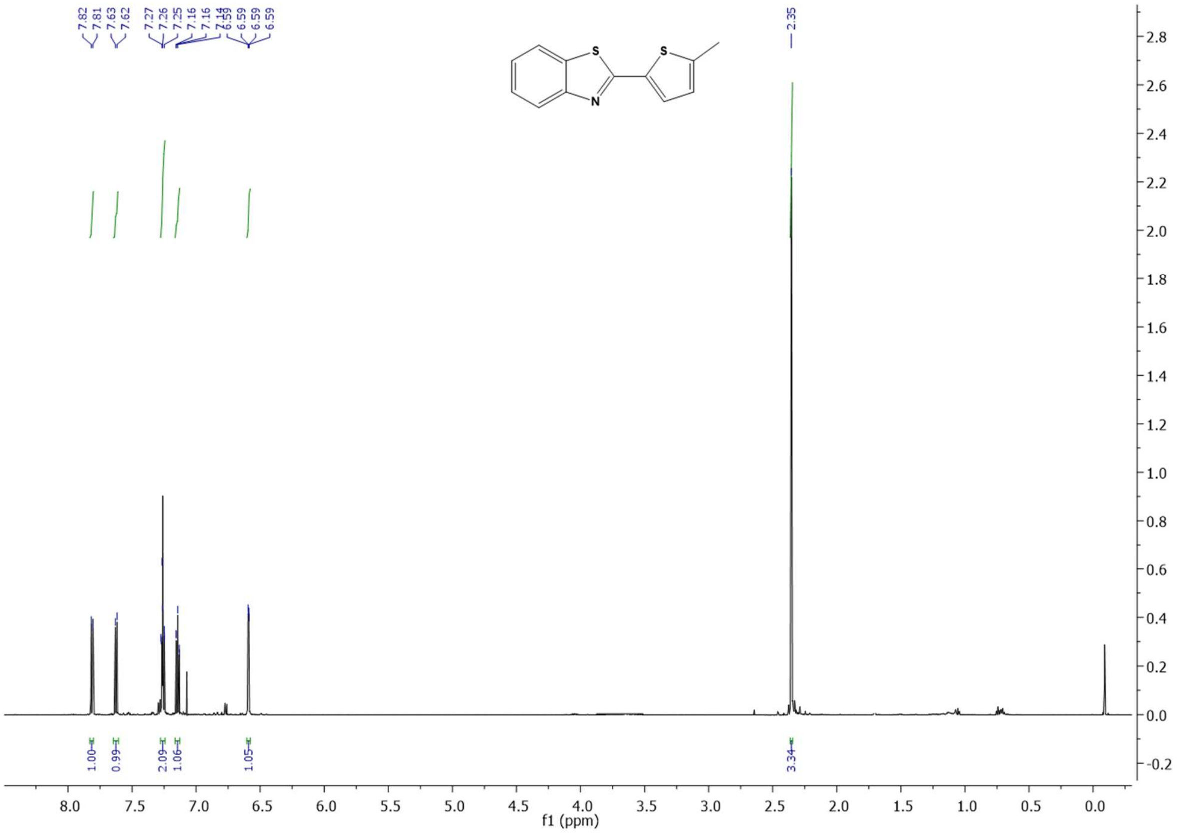

Figure S1: ^{1}H NMR spectrum of 2-(5-methylthiophen-2-yl)benzo[d]thiazole (**3**)

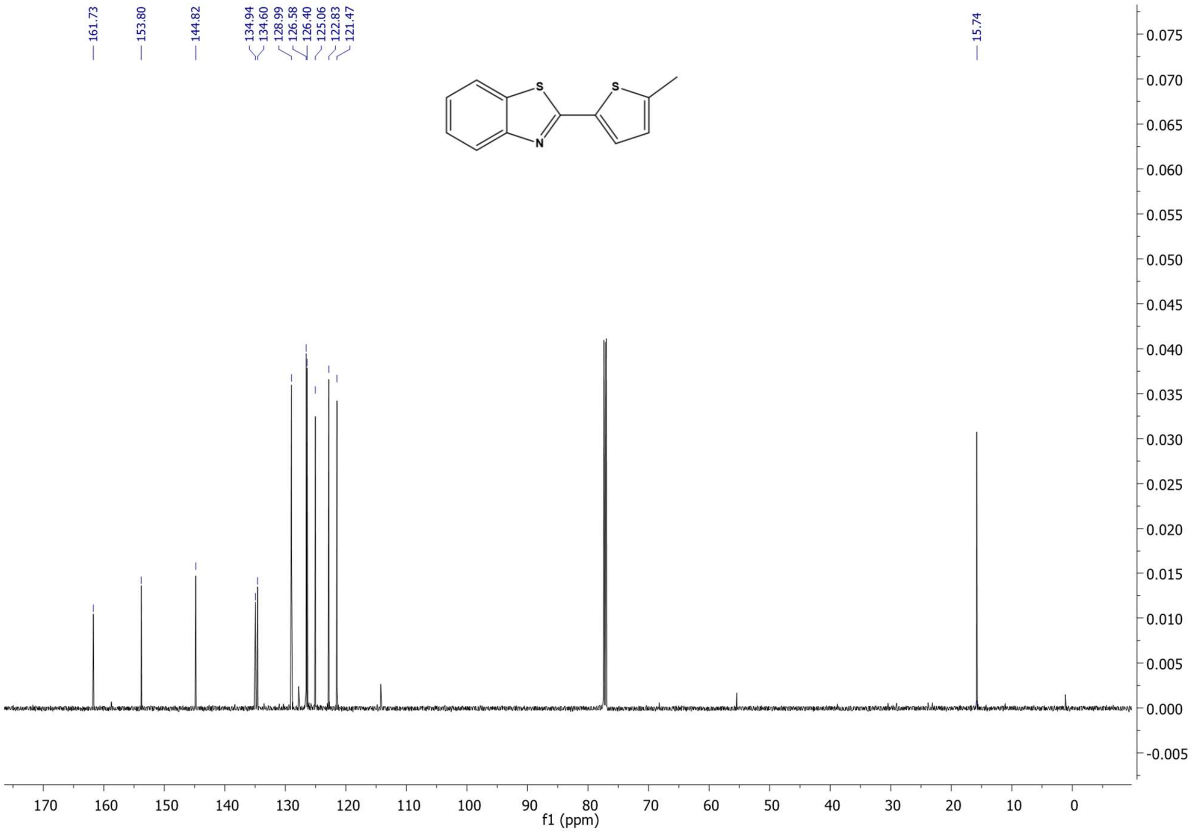

Figure S2: ^{13}C NMR Spectrum of 2-(5-methylthiophen-2-yl)benzo[d]thiazole (**3**)
